# Psychic costs in medical education: do they exist?

**DOI:** 10.11604/pamj.2013.15.67.2937

**Published:** 2013-06-23

**Authors:** Kieran Walsh

**Affiliations:** 1 BMJ Learning, BMJ Publishing Group, BMA House, Tavistock Square, London WC1H 9JR, UK

**Keywords:** Medical education, costs

## Editorial

Imagine the following scenario: You are head of the medical education unit at a large teaching hospital. You have had a contract with an external organisation that provides assessment services, but that contract has just run out and you now need to decide whether to renew or go with an alternative provider. You consider the costs of switching provider but also have less tangible concerns about going with a lower cost service — even though some of the lower cost alternatives seem excellent. You have had personal experience of an assessment system breaking down in the past. You spend a lot of time worrying about the possible consequences of various decisions even though you know that some potential consequences are extremely unlikely. What should you do? And what should you stop doing?

Medical education - like all forms of healthcare professionals’ education - is associated with substantial costs [1]. As a result there has been a growing interest in recent years in ensuring that good value in education is delivered for the costs spent. When writing about the subject myself, I have always dismissed the issue of non-financial costs and concentrated exclusively on financial costs [2]. However some feel that this approach is wrong and that we should also examine psychic costs in education when working up the total costs of educational interventions. Psychic costs relate to the costs of stress when making purchasing decisions [3].

For example a medical educator might decide to purchase a license for online learning resources and might decide to purchase the lowest cost option. But the educator might subsequently feel awkward at having purchased the lowest cost option and might spend time unnecessarily pondering over whether they made the right decision for the right reasons. This is the psychic cost, and if the psychic cost is too high it might outweigh any savings resulting from the lower cost purchase. Psychic costs are purely psychological and should not be confused with other costs which are more tangible such as search costs and switching costs. Search costs relate to the amount of time that a purchaser might spend in searching for perhaps a lower cost option and switching costs relate to the amount of time moving from one provider of education interventions — perhaps an online portfolio — to another. Search costs and switching costs can be applied to a range of different purchases in different circumstances [4].

As well as psychic costs, there may occasionally be psychic benefits that result from purchasing decisions. For example an educator might purchase state-of-the-art video conferencing facilities. The facilities might be expensive but the educator may be thinking of the reduced travel that will result and consequently the reduced carbon footprint. The educator might not allocate a financial sum to the reduced carbon footprint but might nonetheless feel a psychic benefit from the decision to purchase.

Recognising and reflecting upon psychic costs and articulating such reflections is worthwhile as it will enable educators to make more logical and rational decisions and not be distracted by non-financial or psychic considerations. Many sellers are adept at recognising psychic costs and developing strategies to nullify them in the purchaser. So guarantees might allay the psychic cost of worrying that a piece of just purchased education equipment might break down, and exchange policies might allay the psychic cost of worrying that purchasers will be stuck with unsatisfactory equipment. These psychic costs are exactly that - psychic — but the monetary investments required to allay them are all too real. Purchasers should try to make rational decisions and should consider whether investments designed to mitigate psychic costs are really necessary.

However not everyone agrees that psychic costs really exist or that we should take account of them when making decisions. One problem with psychic costs or indeed benefits is that anyone can feel that they have experienced them, and can use them - consciously or unconsciously — to justify decisions. For example an educator could justify paying a high price for an educational intervention as they wished to avoid worry or guilt that they had bought an inferior quality intervention to save costs.

So do psychic costs exist and if so what should we do about them? Undoubtedly psychic costs do exist — just as feelings about any other concept or idea exist. However taking account of them is another matter. Ideally we should reflect on our concerns about a decision that involves cost and articulate those reflections and share them with others. As with a lot of worries, getting them out in the open can go a considerable way to putting them into perspective. However we shouldn't assign a monetary value to them or allow them to help us justify decisions that involve costs. Psychic costs exist — but not on the balance sheet.
